# Arsenic pollution in Quaternary sediments and water near a former gold mine

**DOI:** 10.1038/s41598-020-74403-3

**Published:** 2020-10-28

**Authors:** Łukasz Stachnik, Bartosz Korabiewski, Jerzy Raczyk, Michał Łopuch, Iwo Wieczorek

**Affiliations:** Department of Physical Geography, Faculty of Earth Sciences and Environmental Management, University of Wrocław, Wojciecha Cybulskiego 34, Wrocław, 50-205 Poland

**Keywords:** Environmental chemistry, Hydrology, Natural hazards

## Abstract

Contamination of water and sediments with arsenic and heavy metals is a global issue affecting human health. Regions covered with Quaternary deposits have received little attention from the point of view of the flux of arsenic and heavy metals from sediments to surface water. This study aims to determine the flux of arsenic and other heavy metals from Quaternary sediments to surface waters in an area affected by the former Złoty Stok gold and arsenic mine. Contamination in surface waters and sediments was caused by arsenic, whereas concentrations of metals were usually within water quality standards. Arsenic contamination of surface water increased in the lower part of the basin covered by Quaternary sediments, and exceeded water quality standards by 2 orders of magnitude. Arsenic mass flux exceeded 8 kg/day near the confluence of the Trująca River with the Nysa Kłodzka, a main tributary of the Oder River. An increase in arsenic concentration in the lower part of the basin is related to mine tailings and preferential flow of groundwater through Quaternary sediments. In future, water resources scarcity may lead to an increase in arsenic contamination in surface and groundwater.

## Introduction

Contamination of water and soils with arsenic is an issue in over 70 countries and affects more than 140 million people due to the health risks associated with the consumption of As-contaminated water and food, including carcinogenic effects and keratosis^1–4^. This is usually related to high natural concentrations in groundwater and anthropogenic activity including mining activity^5^ and coal combustion^6^. Elevated arsenic concentrations in groundwater contribute to soil pollution in agricultural areas resulting in the incorporation of arsenic into the food chain^4,7,8^.

Dissolution of arsenic-bearing minerals associated with gold mines and their tailings causes an increase in concentrations of arsenic species in soil and surface water, but the spatial distribution of this pollution is relatively unknown. Studies have focused on the direct impact of mining activity on soil and water properties^7^, whereas less emphasis has been placed on soil and water pollution in larger basins. This appears to be especially important in the case of riverine transport affecting larger areas via solute and sediment-bound yields^9,10^ and ion exchange processes^11^. Moreover, variation in the physiochemical properties of water (e.g. pH, water temperature^12,13^), meteorological conditions (evaporation^12,14,15^), suspended sediment and organic matter transport^14^, water residence time^16^, groundwater withdrawal^17^, and concentrations of phosphate and iron oxides^14,18,19^ affect the seasonal dynamics of arsenic species in surface waters. For example, the summer (as opposed to winter) season, with its high air and water temperatures, leads to an increase in As mass fluxes^12–14^. Seasonal changes influencing arsenic pollution in downstream locations affect water reservoirs, leading to potential human health risks associated with water consumption^7,20^.Water pollution also leads to increased bioaccumulation of arsenic by crops, elevating the risk of food contamination^8^. The development of organic forms of arsenic (e.g. monomethylated and dimethylated acids) enhances arsenic bioaccumulation^21^. Assessment of the spatial distribution of arsenic contamination remains an issue in areas influenced by gold and arsenic mining.

Groundwater flow through Quaternary fluvial and post-glacial deposits usually enhances the release of arsenic species, but their relationship with surface water contamination in these areas requires further studies. The concentration of arsenic species is higher in glacial till than in other types of unconsolidated sediments^22,23^. In a wide variety of Quaternary deposits, the occurrence of intercalated clays and organic carbon results in a higher rate of release of arsenic species into groundwater^24^. For example, palaeochannel deposits consisting of clay and organic rich sediment facies were characterised by arsenic concentrations nearly an order higher than those in a sandy aquifer^25,26^. The long residence time of water in clay-rich Quaternary deposits also facilitates arsenic release to groundwater^27^. A gap exists in knowledge concerning the release of arsenic species from Quaternary aquifers into surface waters.

Previous studies in the Złoty Stok area were characterised by their limited focus on the spatial distribution of arsenic and heavy metal contamination in the lower part of the basin near the confluence of the Trująca River with the Nysa Kłodzka, a main tributary of the Oder River. Sediments and soils in the vicinity of the town of Złoty Stok have been intensively studied from the point of view of contamination^28–30^, biological uptake of As and its remediation^31–36^, and uptake by humans^37,38^. These studies showed very high levels of arsenic contamination in alluvial and anthropogenic soils reaching up to 10.3 10^3^ and 43.5 10^3^ mg/kg, respectively^28^. Surface and groundwater appear to also be contaminated by arsenic, mercury, and manganese upstream of Złoty Stok^39–41^. It is worth noting that the Trująca River may pollute water reservoirs situated on the Nysa Kłodzka River. Even though the contamination of water and sediments near the former gold and arsenic mine in Złoty Stok is well known, knowledge concerning the spatial distribution of contamination in the lower part of basin is lacking.

This paper aims to determine the spatial distribution of pollution from arsenic and other metals in surface waters and Quaternary sediments influenced by the former gold and arsenic mine. Studies dealing with contamination in Quaternary deposits are limited, although water flow conditions in these sediments appear to favour release of these metals into water. Water contamination downstream of the gold mine exhibits a potential risk for contamination in higher-order basins such as Nysa Kłodzka. Earlier studies in the Złoty Stok area did not investigate the contamination of soils and surface water in downstream locations.

## Study area

**Figure 1:**
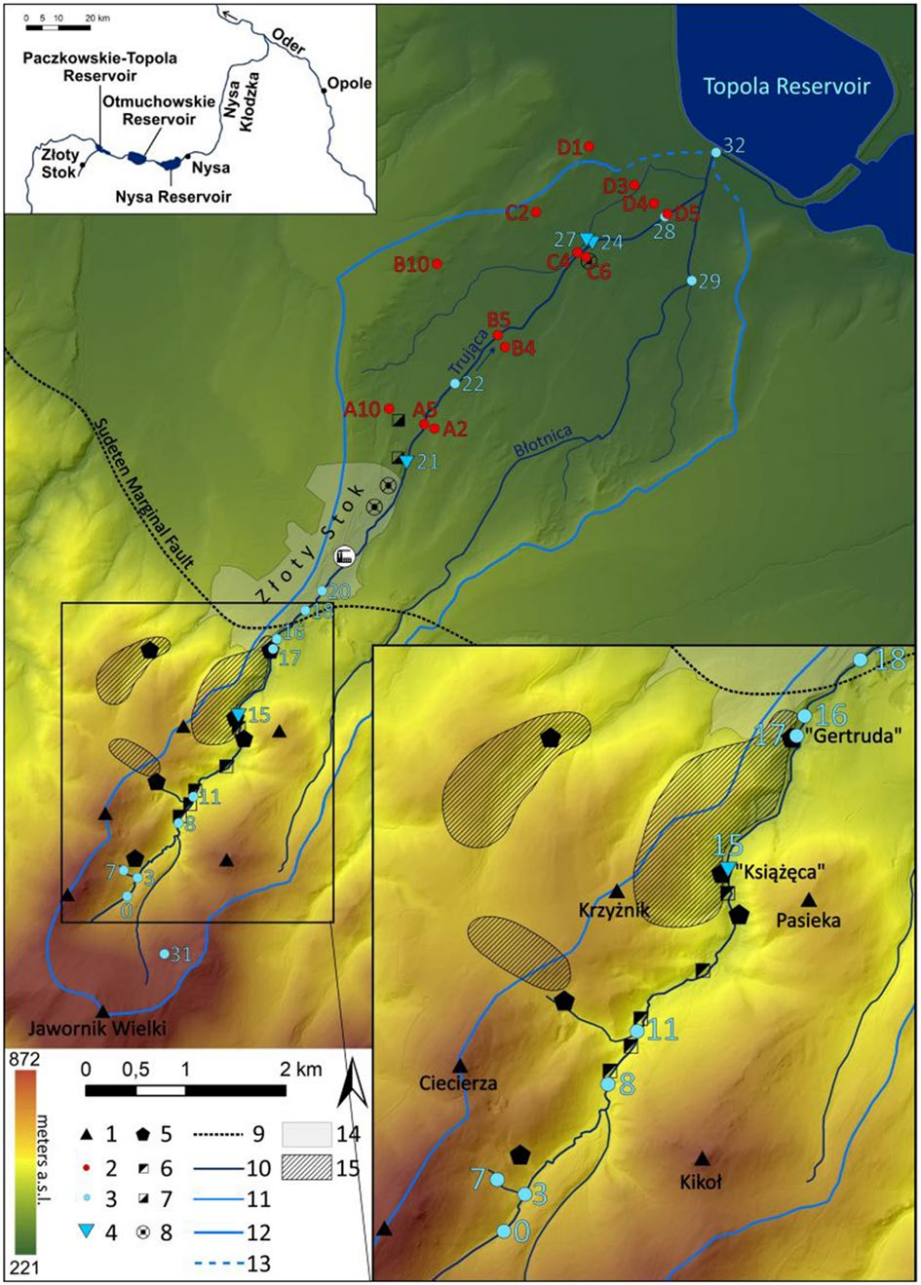
Study area: 1—peak, 2—soil sample, 3—water sample, 4—water gauging station (hydrometric station), 5—shaft, 6—tailings from mine, 7—tailings from dye-work, 8—sedimentation tank, 9—Sudeten Marginal Fault, 10—rivers, 11—rivers (2nd order), 12—watershed boundary, 13—watershed boundary (uncertain), 14—Złoty Stok (town area), 15—major mining areas. Figure generated in ArcMap 10.3 (https://desktop.arcgis.com/en/arcmap/).

The Trująca (roughly translated as “poisonous”) River basin is located on the border of two physiogeographical units, the upper part represented by the Złote Mountains, a part of the Eastern Sudetes, and the lower part by the Otmuchów Depression, a part of the Sudeten Foreland^42^. These parts are separated by the escarpment of the Sudetic Marginal Fault (Fig. 1).

The Trująca River flows into Paczkowskie Lake, established on Nysa Kłodzka, a tributary of the Oder River, as an anti-flood reservoir between 1995 and 2003. The climate is temperate with cold winters and warm summers. Mean annual air temperature is 8 °C and the sum of precipitation ranges between 600 and 700 mm. Snowfalls usually occur between September and March and continuous snow cover is observed for 40–60 days annually^43^. Low river discharge is a typical feature during summer heat waves, whereas high discharge occurs as a consequence of snowmelt in spring and intensive rainfall events in autumn^44^.

**Figure 2:**
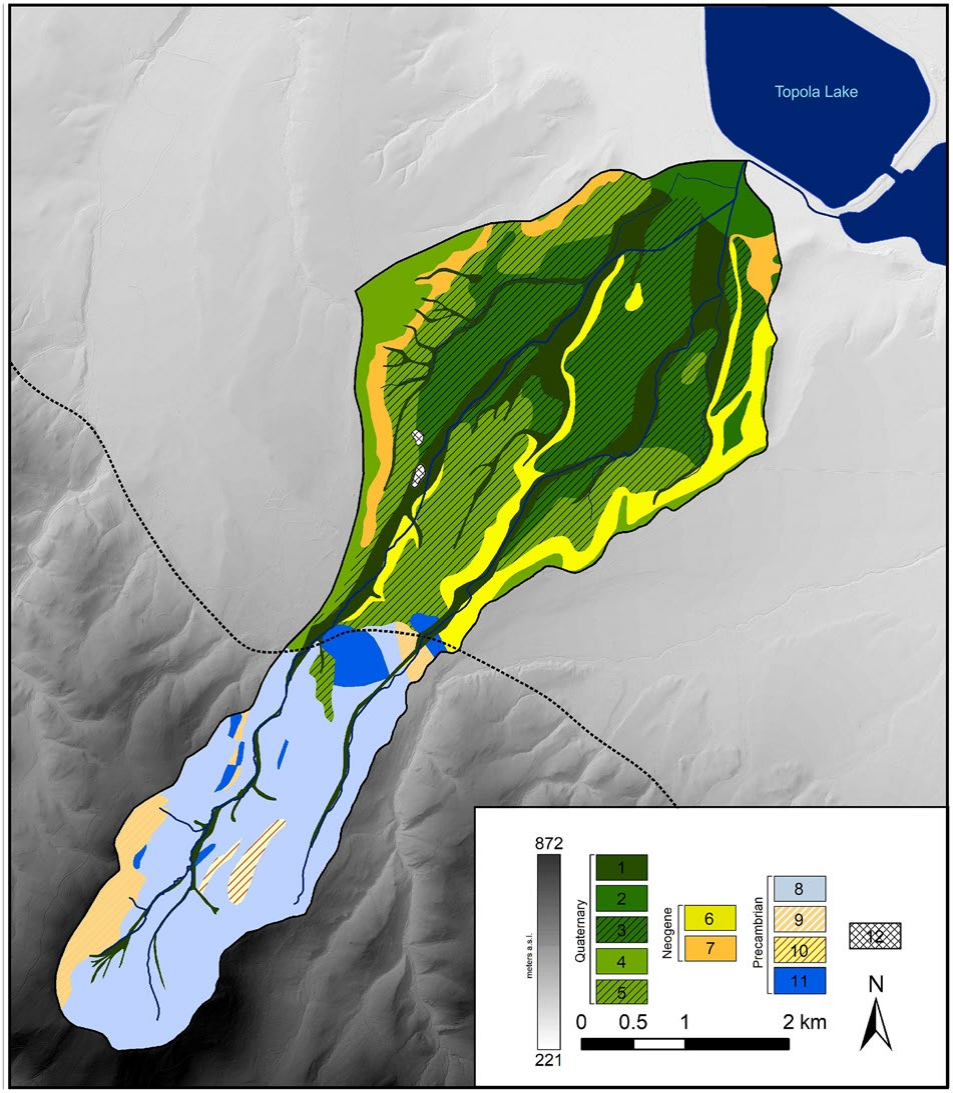
Geological map of the Trująca River basin: 1—fluvial sand and clay, 2—fluvial sand and pebble, 3—fluvial sand and pebbles on Pliocene sediments, 4—fluvial pebbles (high terrace), 5—fluvial pebbles on Pliocene sediments (high terrace), 6—quartzite clay, sand and pebble, 7—clay, 8—schist, 9—gneiss, 10—amphibolite, 11—limestone, 12—mine tailings. Figure generated in ArcMap 10.3 (https://desktop.arcgis.com/en/arcmap/).

The upper and the lower parts of the Trująca River basin differ significantly in terms of geological settings. The upper part is mountainous and built mainly of Precambrian metamorphic rocks (mainly blastomylonitic schist and gneiss) affected by the Skrzynka-Złoty Stok tectonic zone^45–47^ (Fig. 2). The lower part consists of a valley, eroded in Quaternary fluvial sands and gravels and filled with alluvium composed of weathered material from the upper basin. Fluvial units are underlain by impermeable Pliocene silts^48^. The alpine orogenic phase in the upper part of the basin  upper basin led to the migration of thermal solutions and mineralisation of ore minerals, as observed mainly in arsenic-bearing minerals such as löllingite (FeAs_2_) and arsenopyrite (FeAsS), accompanied by magnetite, pyrrhotite and pyrite^49–51^. Secondary arsenic minerals (SAM) consists of Fe arsenates oxidised under acidic conditions or formed by interactions between acidic As-rich solutions and carbonates (e.g. scorodite and pitticite). Other SAMs (e.g., erythrite and annabergite) crystallised from slightly acidic to neutral pHs^52^.

Gold and arsenic were the most important resources from the Złoty Stok mines and were exploited from the thirteenth century until the closing of the last mine in 1961^53,54^. The landscape of the upper part of the basin was changed drastically during several hundred years of mining activity. As many as ten large mine tailings are located on the banks of the Trująca, constituting a possible source of river contamination (Fig. 1). Since 1962, a dye factory (Złoty Stok Grupa S.A.) has operated in Złoty Stok^55^, making use of two sedimentary tanks and wastes stored on the tailings located in the vicinity of the Trująca River (Fig. 1).

## Results

**Figure 3:**
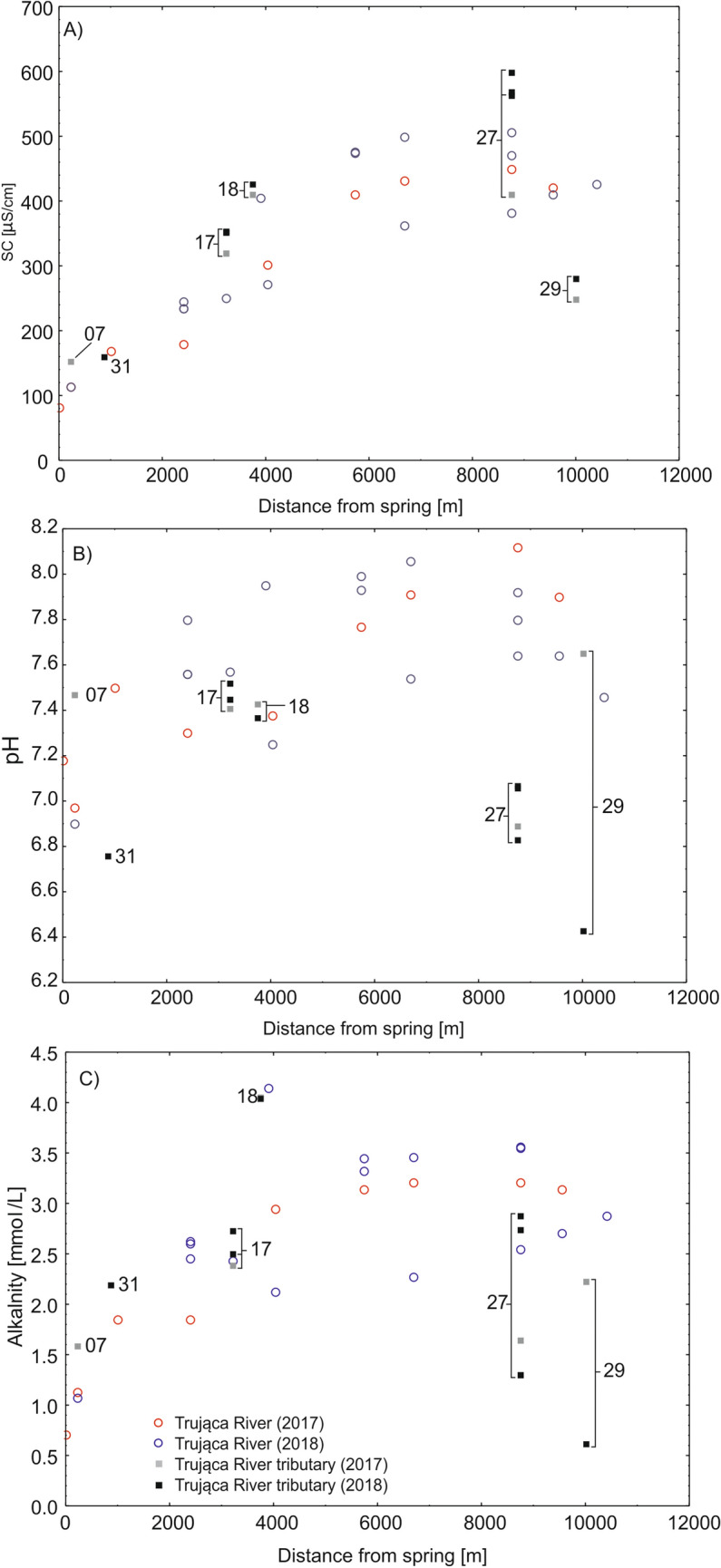
Physiochemical properties of surface and groundwater in the Trująca River (circles) and its tributaries (rectangles) in 2017 and 2018: (**A**) water specific conductivity (SC), (**B**) pH, (**C**) alkalinity. Detailed locations of sites are marked in Fig. 1.

SC, pH, and alkalinity increased from the basin head to points downstream of the Trująca River basin. The basin head was characterised by SC as being below 250 µS/cm, a value which increased twofold in downstream locations. Groundwater, including the samples from Gertruda and Ochrowa adits (nos 17 and 18, respectively; Fig. 1) had slightly higher SC values. In downstream sites, the SC of the stream draining the agricultural basin (no. 27) was the highest compared with other points; however, the small stream (no. 29; Fig. 1) was characterised by much lower SC, falling below 300 µS/cm (Fig. 3A). Alkalinity generally showed a pattern similar to that of SC, varying from ca 1–1.5 mmol/L in the head basin to nearly 3.5 mmol/L in downstream locations (Fig. 3C). Nevertheless, concentrations lower than or similar to that of the main stream were noted for agricultural streams (nos. 27 and 29; Fig. 1). Values of pH changed from circumneutral values of the main river in the head basin (pH 6.9–7.2) to marginally alkaline values in downstream sites (pH 7.8–8.2; Fig. 3B). The pH of groundwater was similar to that of the main stream (pH ~ 7.5), but was circumneutral for agricultural streams (Fig. 3B).

**Figure 4:**
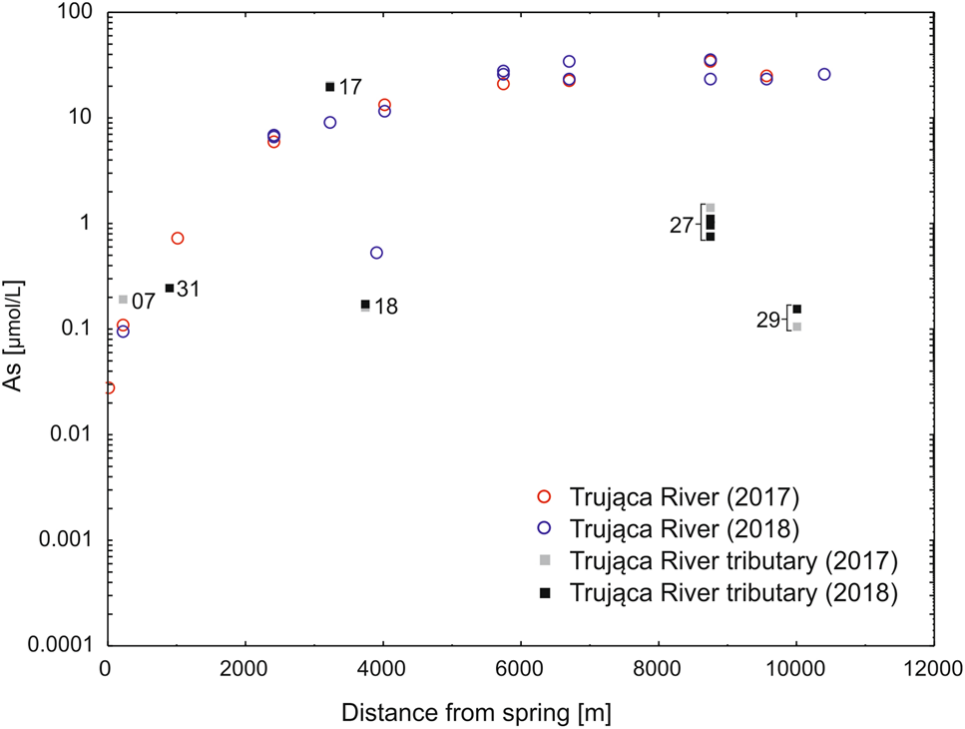
Arsenic concentration in surface and groundwater in the Trująca River (circles) and its tributaries (rectangles) in 2017 and 2018. Detailed locations of sites are marked in Fig. 1.

**Figure 5:**
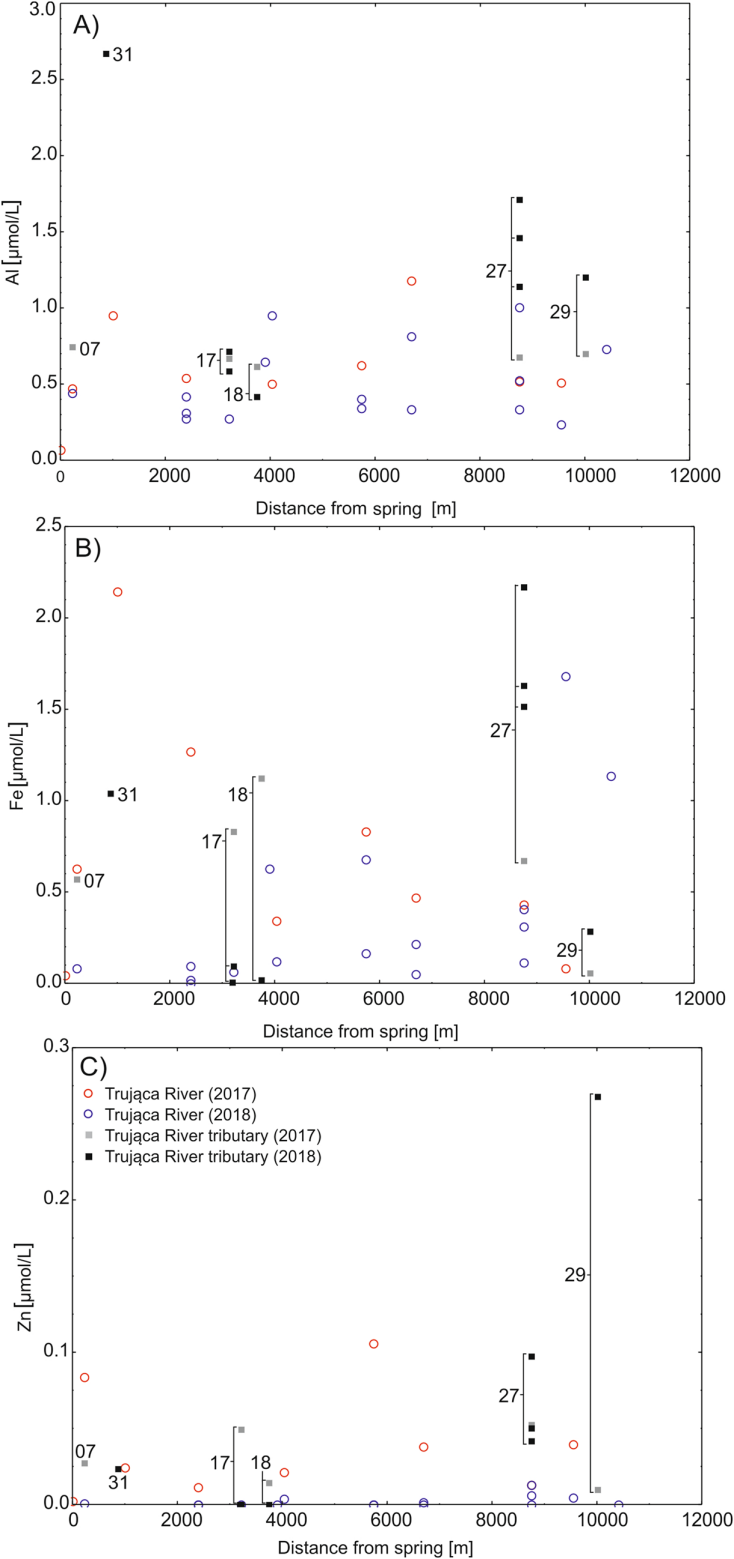
Al, Fe and Zn concentrations in surface and groundwater in the Trująca River (circles) and its tributaries (rectangles) in 2017 and 2018. Detailed locations of sites are marked in Fig. 1.

Only arsenic concentration showed a clear increase from the head basin to downstream locations, whereas concentrations of other metals remained stable. In the head basin, the concentration of arsenic in surface waters did not exceed 1 µmol/L. In downstream locations below the dye factory, there was a pronounced rise in arsenic concentration from 8.5 to 32.9 µmol/L. At the river mouth, arsenic concentration measured 15 µmol/L (Fig. 4). Concentrations of arsenic were usually higher in groundwater (e.g. no. 17; Fig. 1), exceeding 1 µmol/L, than in surface waters. In agricultural streams unaffected by the Trująca River (nos. 24 and 27; Fig. 1), arsenic concentrations were, on average, below 0.8 µmol/L, but, nevertheless, above water quality standards. Concentrations of Zn, Fe and Al were stable along the course of the Trująca River and in agricultural streams (Fig. 5) and generally appeared not to exceed 0.1, 2, and 2 µmol/L, respectively (Fig. 5A–C).

**Figure 6:**
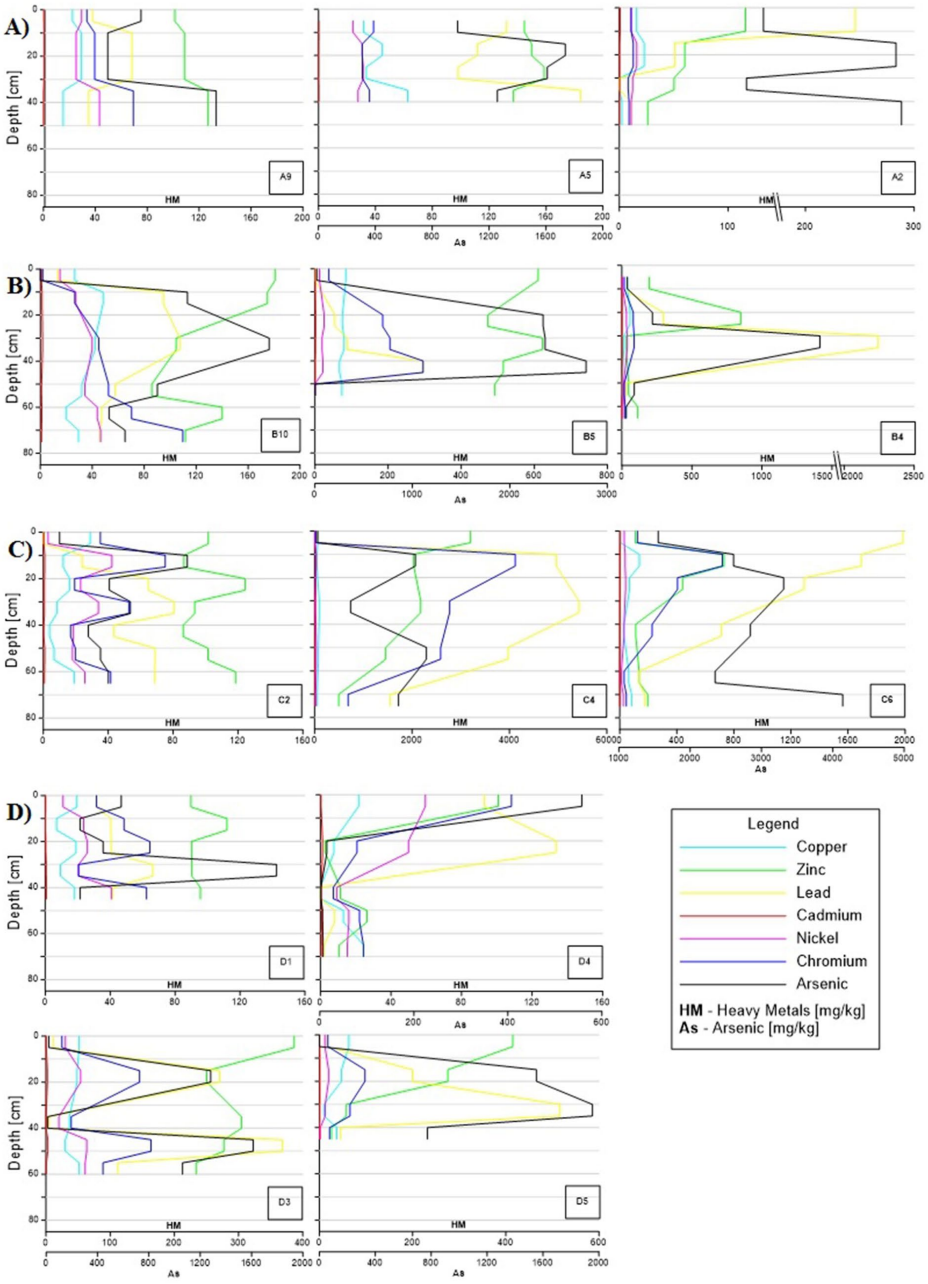
Heavy metals (Cu, Zn, Pb, Cd, Ni, Cr) and arsenic contents in mg/kg in sediments collected from four transects in the Trująca River basin: (**A**) transect A (A9, A5, A2); (**B**) transect B (B10, B5, B4); (**C**) transect C (C2, C4, C6); (**D**) transect D (D1, D3, D4, D5). The position of the sites are shown in Fig. 1.

The concentrations of As and heavy metals (Cu, Zn, Pb, Cd, Ni, Cr) exhibited very large differences in profiles and between transects (Fig. 6). Arsenic-polluted sediments dominated in alluvial deposits, whereas sediments in the valley slopes were much less polluted. Profiles situated on the valley slopes (nos. A2, A9, B10, C2, D1, D4; Fig. 1) showed relatively low As content, usually below 160 mg/kg dw, excluding several samples with higher values (surface samples in D4 and in the middle part of A2). The other profiles represented alluvial deposits in which As concentrations usually exceeded 1000 mg/kg (nos. A5, B4, B5, D3, D5; Fig. 1), reaching up to 4000 mg/kg dw locally (nos. C4, C6; Fig. 1).

**Figure 7:**
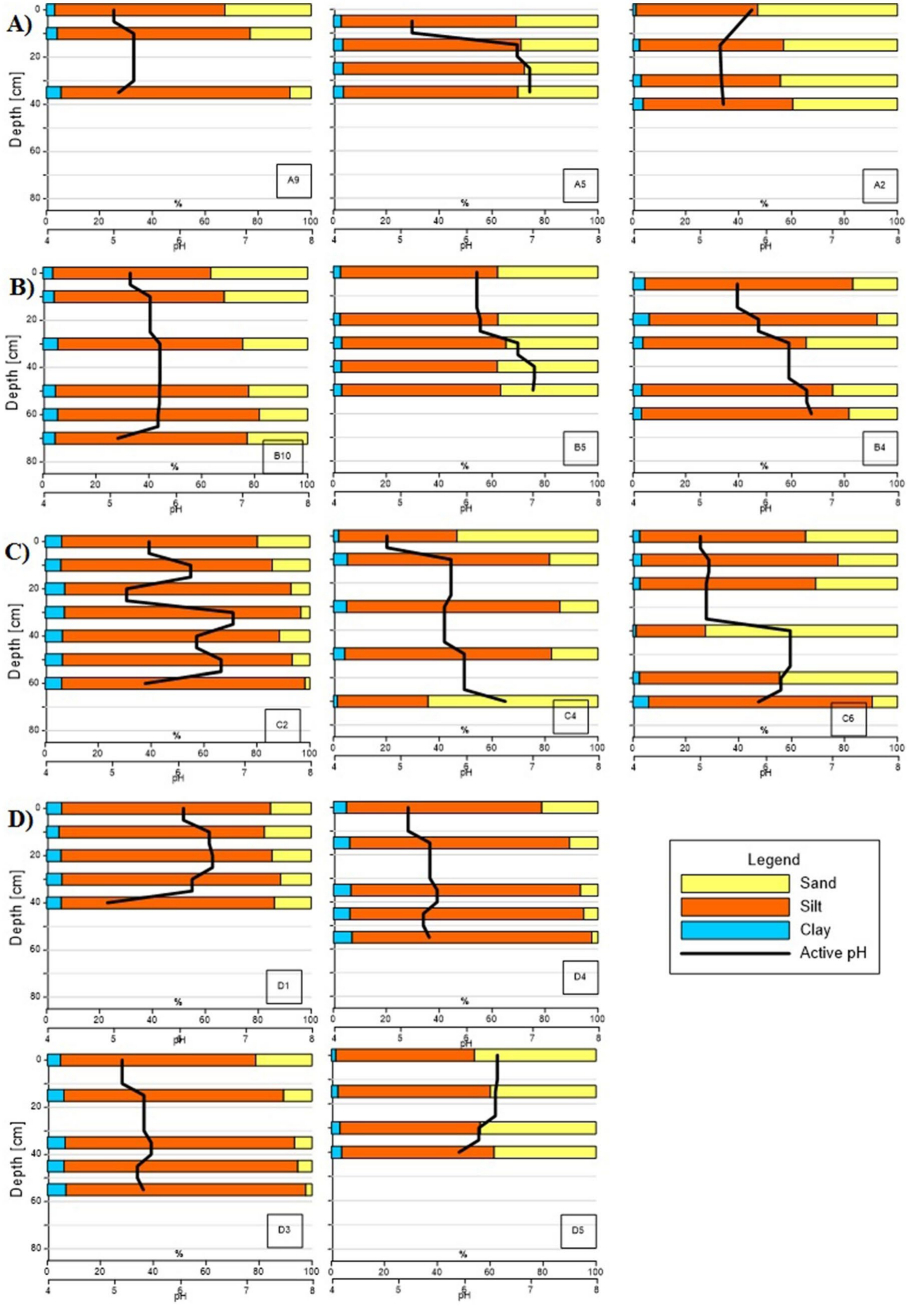
Granulometric fraction and pH of sediments in the Trująca River basin in the following transects: (**A**) transect A (A9, A5, A2); (**B**) transect B (B10, B5, B4); (**C**) transect C (C2, C4, C6); (**D**) transect D (D1, D3, D4, D5). The position of the sites are shown in Fig. 1.

Sediments on valley slopes were usually characterised by a systematic increase in sand fraction towards the top of soil profiles. In the sediment fraction the dominant element in the fine earth fraction (< 2.0 mm) was mainly silt (0.002–0.063 mm). Its average content in alluvial sediment was 65%, in slope sediment 69%. Sediments in the bottom of the valley were characterised by fraction diversity but were not regular in vertical profiles (Fig. 7A–D). At the same time, alluvial sediments were characterised by higher contents of sandy fraction. The clay fraction was on average 4.1% (0.8–7.4%) for all samples, whereas alluvial sediments were characterised by slightly lower clay contents.

The pH_H2O_ in alluvial sediments increased with the depth of the sediment profile, with the lowest value usually found in the surface levels enhancing As leaching. A difference between the slopes and alluvial sediments is also observed. Alluvial sediments usually exhibited a higher pH, ranging from pH 4.8 to 7.0 (on average pH 5.6), compared to sediments on the valley slopes (average pH 4.6, range pH 3.6–6.8).

## Discussion

This study provides multidisciplinary data on the distribution of arsenic and heavy metals in Quaternary sediments and surface waters in a heavily polluted area affected by gold and arsenic mining activity. We show the relationship between sediment and water contamination in a small catchment of the Trująca River situated in the upper part of the basin of the Oder, the second largest Polish river.

In the upper part of the basin, concentrations of arsenic and heavy metals (especially Fe, Zn) obtained in earlier studies were one or more orders of magnitude higher than in our surface and groundwater samples . For example, arsenic concentrations in the Gertruda adit were higher by a factor of ~ 27 in 1996^56^ and ~ 6 in the 2010s^41^, whereas in the Ochrowa adit, arsenic concentrations were higher by a factor of ~ 180 between 2012 and 2014^40^. Average concentrations of Fe and Zn from 1996 and the 2010s in the Gertruda adit were lower by factors of ~ 4 and ~ 46, respectively, compared to our results.

Heavy metal and arsenic concentrations in surface waters were usually much lower compared with earlier studies. In surface waters (nos. 16, 08; Fig. 1), arsenic concentrations in our samples were lower by factors ranging from ~ 50 to ~ 360 compared with analyses from 1996^56^. In our hydrochemical data, concentrations of Zn in surface water were approximately 1 to 2 orders of magnitude higher when compared to results from 1996^56^. Only the concentration of Al was higher in surface water in our study compared to earlier studies^41,56^. The physiochemical properties of water and metals (As, Fe) concentrations at one site (near the Ochrowa adit, Fig. 1) according to long-term records from 2011 to 2017 collected by the Voivodeship Inspectorates for Environmental Protection in Wrocław (WIOŚ in Wrocław) were similar to those found in data from the upper part of the basin^57–63^. Concentrations of Zn were lower compared with data from WIOŚ in Wrocław. Decreases in As and Zn concentrations from 1990s to 2017–2018 may be attributable to a lowering in aquifer depth, remediation activities in the mine, or possible differences in the position of sampling points.

The great discrepancy in the concentration of arsenic and heavy metals in the upstream locations appears to be related to changes in the hydrological regime. Discharge in the Trująca River at sites situated upstream of the town of Złoty Stok has decreased considerably from 20 L/s and 43–72 in in 1990s^56,64^ to less than 1 L/s in November 2018 (Table 2). In 2018, discharge of surface water was equivalent to discharge from springs in the headwaters of the upper part of the Nysa Kłodzka basin^65^. Stream flow dominated by baseflow in the Trująca River basin^64^ and the falling groundwater table in the Sudetes Mts. in the period 2002–2015 led to reduced surface water discharge^66^.The decrease in groundwater flow is evident in the 2018 discharge data and reflects the falling aquifer level. This leads, in turn, to reduced water content in surface sediments, including polluted sediments, sludge and mine tailings, which likely inhibits the dissolution of arsenic-bearing minerals and thus the leaching of arsenic and other metals from groundwater into surface waters. In the Złoty Stok mine, renovation activities, including the removal of mine tailings and clearing of adits or improvement of mine drainage, also facilitate reduction of the residence time of water.

### Soil contamination downstream

The contents of heavy metals, excluding arsenic, in the majority of soil samples are below Polish levels defining soil contamination^67^. Regardless of land-use category, arsenic concentration exceeds the Polish level of soil contamination by up to two orders of magnitude. Most of the profiles (9) were assigned to soil group III including forests (As > 50 mg/kg), whereas the rest were assigned to arable land (2) and grassland (2), which belong to group II-2 (As > 20 mg/kg). The content of arsenic in soils between transect A and the dye factory^68^ was as high as ~ 1000 mg/kg; this was much lower than our results, which showed a systematic increase from ~ 1400 mg/kg (e.g. nos. A5, B5; Fig. 1) to ~ 3000 mg/kg in downstream locations of the Trująca River valley (e.g. nos. C6, C4; Fig. 1); (Fig. 6). Similar and higher values (> 6000 mg/kg) were also observed in soil samples collected between transects A and B^28,30^.

Mobile arsenic species, non-specifically (extracted by 0.05 M (NH_4_)_2_SO_4_) and specifically (extracted by 0.05 M NH_4_H_2_PO_4_) sorbed, constitute for less than ~ 2%, and 20%, respectively for alluvial soils situated between transects A and B^28,69^. Less mobile arsenic species bonded to amorphous and poorly-crystalline Al and Fe oxides exceed 30% of total As and are usually higher than poorly bioavailable As bond to well crystallized Fe and Al oxides^28,69^. Previous studies also show the significant impact of changing redox conditions on arsenic mobilisation due to Fe and Mn reduction and reductive dissolution^38^. In acidic (pH < 5.0) conditions and anaerobic experiments at initial alkaline pH (pH = 8.0) As solubility increases leading to As release in alluvial soil^28^. Although anaerobic conditions may develop in the lower part of the Trująca River basin due to boggy terrain (transects C and D; Fig. 1), pH values, usually below 6.0 inhibit As release. On the other hand, at a downstream location at the Trująca Valley bottom (points D3, D4, C4), species characterised by acidic soil pH also favour the release of As into pore water and streams (Figs. 6C,D; 7C,D). As leaching from As-bearing Fe and Mn oxides likely depends on the activity of autochthonous bacteria, either As-resistant or dissimilatory-As(V) reducing^70^.

Arsenic content varies greatly in vertical sediment profiles. In general, most of the profiles show reduced As content at the surface level and increased content in the middle or bottom of the sediment profile. This tendency was observed both in heavily polluted sediments at the bottom of the valley and the less polluted slopes making it difficult to distinguish a single factor affecting the distribution of sediment contamination. This great variation may result from marked anthropogenic transformation including changes in the course of the river, embankment construction at the valley bottom, and agricultural activity (e.g. fields of crops) on the valley slopes.

### Water contamination downstream

The chemistry of surface waters and the physiochemical properties of water in the lower part of the Trująca River basin are similar to those in studies carried out in 2010–2017 by the Wrocław WIOŚ. In the lower part of the basin, where arsenic concentration reached its highest values (Table 1, Fig. 4), the initial data on metal concentrations were obtained for one point in 2017 by the Wrocław WIOŚ (near no. 18; Fig. 1) were similar to our results^63^. Moreover, the physiochemical properties of water (pH, SC) from our study were comparable with multi-annual data from the Wrocław WIOŚ^57–63^.

**Table 1:** Specific conductivity (SC, in µS/cm), alkalinity (mmol/L), pH, and heavy metal concentration in µmol/L in water collected in the Trująca River basin.

Site	Parameter	SC	pH	Alk	As	Fe	Al	Zn
Upper	Mean	195	7.24	2.1	3.2	0.55	0.65	0.0145
	Median	174	7.48	2.0	0.6	0.33	0.46	0.0016
	Min–Max	81–406	6.76–7.95	0.7–4.1	0–9.3	0–2.15	0.07–2.67	0–0.084
Mine	Mean	372	7.43	3.1	12.3	0.42	0.60	0.0128
	Median	353	7.43	2.7	19.9	0.09	0.62	0.0001
	Min–Max	320–427	7.37–7.52	2.4–4.1	0.2–21	0.02–1.12	0.42–0.71	0–0.0495
Lower	Mean	420	7.68	3.0	26.0	0.47	0.60	0.0164
	Median	426	7.80	3.1	25.4	0.34	0.52	0.0048
	Min–Max	272–506	7.25–8.12	2.1–3.6	11.7–36	0.05–1.68	0.24–1.18	0–0.106
AG	Mean	445	6.85	1.9	0.76	1.05	1.15	0.0867
	Median	487.5	6.97	1.9	0.87	1.09	1.17	0.0513
	Min–Max	249–598	6.43–7.65	0.6–2.9	0.1–1.4	0.05–2.17	0.68–1.71	0.0103–0.2681

The following sites were sampled: the upper part of the Trująca River (nos. 00, 03, 07, 08, 15, 31, 33, 34), the mine (17, 18), the lower part of the Trująca River (20, 21, 22, 24, 28, 32) and agricultural streams (AG) in the lower part outside the Trująca River (27, 29). The position of the sites are shown in Fig. 1

*AG* agricultural streams, *Alk.* Alkalinity.

In the lower part of the basin, arsenic concentrations increased along the Trująca River posing a potential risk for water quality in the upper part of the Nysa Kłodzka basin. Our results show arsenic concentrations two orders of magnitude in excess, of water quality standards for potable water (0.1 µmol/L^71^) and protection of aquatic life (0.07 µmol/L^72,73^); (Fig. 4, Table 2). These elevated concentrations were mainly observed in the lower part of the basin at the confluence of the Trująca River and Paczkowskie Reservoir situated on the Nysa Kłodzka River, a left tributary of the Oder River. Lack of regular monitoring of metal concentration in the Nysa Kłodzka downstream of the confluence with the Trująca River makes it difficult to determine the spatial distribution of arsenic water contamination. Arsenic concentration potentially increased at least fourfold from the Topola Reservoir (< 0.03 µmol/L in 2012^58^) to the Otmuchów (0.16 µmol/L in 2015^74^) and Nysa (0.13 µmol/L in 2015^74^) Reservoirs situated ~ 11 and ~ 22 km, respectively, downstream of the junction of the Trująca and Nysa Kłodzka Rivers (Fig. 1). Moreover, arsenic concentration in the Trująca River appears to be much higher compared with others small rivers (below 0.02 µmol/L) in the Sudetes Mts.^75^.

**Table 2:** Discharge and As mass flux in selected sites in the Trująca River basin in 2018. The position of the sites are shown in Fig. 1.

Site	Description	Q [L/s]	Daily mass flux of As [kg/day]
15	Upper part	0.9	0.04
21	Lower part	31.8	5.76
24	Lower part	35.4	8.25
27	Lower part (agricultural streams)	12.7	0.08

### Mechanisms of arsenic release

Mechanisms of arsenic release vary, from reductive dissolution or sulphide oxidation in the groundwater in the upper part of the basin to alkali desorption in the lower part of the basin. Groundwater in mine adits was characterised by very high concentration of reduced As species (As (III)^41^), high concentrations of other metals (e.g. Fe, Mn^56^; Table 1, Fig. 5), and high alkalinity (Fig. 3). These conditions suggest two processes of arsenic release including reductive dissolution or sulphide oxidation^2,23^. Both processes mediated by the microbes were noted in the in close proximity to each other on microbial mats in the Gertruda adit^41^. Dissolution of crystalline limestone mineralised with arsenopyrite and pyrite drives water pH towards neutral values^76^ suggesting that reductive dissolution appears to be widespread in mine water. On the other hand, downstream changes in arsenic release mechanisms were observed in surface waters, where As concentration rose gradually in spite of stronger oxidation related to prolonged contact with the atmosphere^57–63^. Alkali desorption, a mechanism for the release of oxidised arsenic species (e.g. As(V)) from iron hydroxyoxides and clays^77,78^, may be responsible for arsenic release in surface water^79^. Despite water pH slightly lower than optimal for alkali desorption (pH > 8.0^78^), the elevated concentration of phosphate (~ 70 µM) in surface water^57,59,61^ likely shift alkali desorption towards a slightly lower pH^78,80,81^. Moreover, the mean and median annual pH of surface water for 2011, 2013, 2015 and 2017 (near site no. 27) was ~ 8.2, within a range from 7.8 to 8.8^57,59,61,63^, suggesting that alkali desorption is possible. The occurrence of alkali dissolution in the lower part of the basin suggests that arsenic release increases with increasing phosphate concentration and water pH above 8.0.

The arsenic contamination of the Trująca River not only affects the basin and its surroundings but also exerts a regional impact on water chemistry. Water quality in the Trująca River is among the poorest in the Lower Silesia Voivodeship due to arsenic contamination^82^. However, it is difficult to quantify the impact on water quality and sediment geochemistry in the downstream reservoirs (e.g. Paczkowski, Otmuchów) on the Nysa Kłodzka River directly affected by the Trująca River. It is noteworthy that dissolved arsenic mass flux appears to be as high as 8 kg/day during the low discharge conditions observed in November 2018 (Table 2). During snow melt or extreme rainfall river discharge and suspended sediment reach peak values in the Nysa Kłodzka River^83,84^, increasing the potential for elevated arsenic mass flux, in both dissolved and sediment-bound forms^9,21,85^. Particularly, sediments contaminated with arsenic (> 1000 mg/kg; Fig. 6) originating from the erosion of crop areas in the lower part of the Trująca River basin likely enhances the total  mass flux of arsenic.

### Regional importance of arsenic contamination

The increase in water scarcity caused by droughts may result in greater domestic use of As-contaminated water from the Trująca River basin. In nineteenth and at the beginning of the twentieth century, inhabitants of Złoty Stok and the vicinity developed a wide range of detrimental health conditions, known as Złoty Stok disease (in German *Reichensteiner Krankheit*), caused by drinking As-contaminated water from the Trująca River^86^. These conditions, including dyspepsia, obstipation, nervousness, browning of the skin, and hyperkeratosis, developed until a pipeline was constructed in 1928 to supply less-contaminated water from another part of the Złote Mts^86^. Nowadays, water is mainly supplied via waterworks from the upper part of the basin near Jawornik Wielki (Fig. 1)^87^ with an additional water pipe (since 1978) from Kamieniec Ząbkowicki situated 9 km north of Złoty Stok^88^. The former supplies the town with water during the part of the season characterised by high water demand for example during low groundwater levels. Furthermore, increased water scarcity, as shown by a discharge decrease in the Trująca River (Table 2) or an overall drop in Sudetes spring discharge^65,66^, likely results in water rationing in Złoty Stok. Additional use of As-contaminated groundwater from public and private wells in Złoty Stok area may be required to meet long-term water needs. This process may be occurring in allotment gardens in Złoty Stok, where the soil is characterised by a very high level of As contamination^37^, likely due to the use domestic wells^88^ to water plants. Furthermore, enhanced evaporation and microbial activity, as a result of higher air temperatures, facilitate increases in As concentration in surface water^12,13^ and increase groundwater contamination^17^. Both processes, causing water scarcity, domestic use of water and a reduction in groundwater resources, lead to higher health risks for the inhabitants of the Złoty Stok area due to contamination of water with As.

A high level of contamination of sediment with As in the lower part of the basin suggests the bioaccumulation of this element in croplands, leading to the introduction of high concentrations of As into the food web. Fertilisation in croplands via phosphate and sewage sludge enhance the mobility of As species in soils^89,90^. The valley bottom is characterised by pronounced contamination with As at great distances from the mine (D3, D5, Fig. 6). Consequently, in croplands, bioaccumulation of As in plant shoots and roots of dry fodder has been found to exceed permissible content levels for animal feed^91,92^. Moreover, As content (72–451 mg/kg) in soils in allotment gardens suppling inhabitants of Złoty Stok with fruits and vegetables markedly exceeds soil quality limits^37^. High levels of bioaccumulation over a wide range of crops may affect the inclusion As in food consumed by inhabitants and domestic animals^15^. Additionally, in forested areas surrounding croplands, decomposition of forest litter leads to an increased share of highly mobile As species^93,94^ increasing their transfer to the surrounding arable lands.

A reduction in water resources and human activity in croplands likely leads to a higher risk to human health in the future. In the town of Złoty Stok, the total 5-year number of new cancer incidents per 1000 people (12–22) was rather low as compared with the other gminas in the county of Ząbkowice Śląskie in 1986–2015 (Supplementary Figure S1). This number was similar to small villages situated outside As mining sites (e.g. Ciepłowody, Stoszowice) and was lower by a factor of ~ 2 than in towns in the county (e.g. Kamieniec Ząbkowicki, Ząbkowice Śląskie) (Supplementary Figure S1). High levels of water and sediment contamination will likely increase in future, in association with droughts and increase in cropland areas. For example, arsenic water contamination in the lower part of the basin in Trująca River and agricultural streams exceeds low to moderate concentrations (10–150 µg/L), at such concentrations, higher risks of diseases such as cancers, cardiovascular diseases, respiration problems, and diabetes mellitus were noted^95–98^. Consequently, the risk of cancer will likely increase due to higher consumption of polluted surface and groundwater and crops with high As bioaccumulation.

Future work in the vicinity of Złoty Stok should focus on determining arsenic  mass flux in both organic and inorganic species and the role of soil erosion and water runoff on arsenic transport from the Trująca to the Nysa Kłodzka River. So far, no analyses of inorganic (As(V), As(III)) or organic arsenic species (associated with monomethylated and dimethylated acids) have been conducted in the surface water of the Trująca River basin. Studies on bioavailable arsenic in soils and its potential release are limited to the immediate vicinity of Złoty Stok and the former gold mine^28,31,41^. Studies in the lower part of the basin are lacking; sequential extraction of arsenic species^28,99^ should be conducted at these sites. Moreover, annual monitoring of water discharge should be performed to determine the mass flux of arsenic species.

In this study we focused on the contamination of surface water and Quaternary sediments in the Trująca River basin, a headwater of the basin of an Oder tributary, i.e. the Nysa Kłodzka River. We have found that As contamination in soils and surface water extends over great distances from the former arsenic and gold mine, producing a negative environmental effect on the upper part of area drained by the Nysa Kłodzka River.

Our study emphasises the high levels of contamination with arsenic species in areas covered by croplands in the lower part of the Trująca River basin. The processes of arsenic release vary, from reductive dissolution in the upper part to alkali desorption in the lower part of the basin, leading to the highest arsenic contamination in the basin, exceeding surface water quality limits by at least two orders of magnitude. Moreover, As contamination of soils and mass flux in surface water (~ 8 kg/day) make this area hazardous in terms of regional-scale contamination (e.g. croplands in the Nysa Kłodzka River and As accumulation in anti-flood reservoirs therein). The increased scarcity of water resources has resulted in As contamination in surface water, leading to a greater supply of this element to groundwater in the lower agricultural part of the basin. Furthermore, high levels of bioaccumulation in croplands has caused introduction of As into the food web, increasing consumption by human and domestic animals. This will likely lead to increased health risks (e.g. cancer) in spite of the number of cancer incidents in Złoty Stok is on average at present. Further studies should be conducted on regional arsenic contamination of the other basin in the upper part of the Nysa Kłodzka basin as manifested by high levels of contamination in the Trująca River.

## Methods

Water samples were collected from the mine (nos. 17 and 18; Fig. 1), the upper (nos. 00, 03, 07, 08, 15, 31, 33, 34; Fig. 1) and lower (nos. 20, 21, 22, 24, 28, 32; Fig. 1) parts of the Trująca River, and agricultural streams (nos. 27 and 29; Fig. 1) in the Trująca River basin. The agricultural streams are tributaries of the Trująca River situated in the lower part of the basin representing contamination outside mine drainage. In total, 38 water samples were collected in November 2017 and October and November 2018. Sampling in both years was chosen on the basis of different meteorological conditions including higher annual sum of precipitation in 2017 (851.8 mm) than in 2018 (710.9 mm) as observed in Lądek Zdrój, town situated 10 km to the south from Złoty Stok^100^. The water samples (referred hereafter as filterable fraction) were immediately filtered through a cellulose acetate syringe filter with a pore size of 0.45 µm^101^. The filtered samples were transferred to HDPE bottles, acidified (Suprapur 30% HCl, Merck), and stored in darkness at 4 °C.

Quaternary sediments were collected from 13 profiles in November 2017, located along four transects perpendicular to the valley axis (Fig. 1). They represent alluvial and slope sediments, both potentially polluted by material from the mine and mine tailings.

### Physiochemical properties of water

In the field, water temperature was analysed using a HI9828 portable thermometer (Hanna Instruments). The accuracy and resolution of water temperature measurements were 0.15 and ± 0.01 °C, respectively. Water samples were analysed for physiochemical properties (pH, and specific conductivity, hereafter SC) in the laboratory of the Department of Physical Geography (University of Wrocław, Poland) immediately following sampling. Measurements were performed using methodology described in the USGS manual^﻿102﻿^. In the laboratory calibration was performed using high-quality pH (pH values of 4.01, 7, and 9.21 Hamilton Company) and SC buffers (100 and 300 µS/cm, Reagecon).

### Discharge measurements

In order to determine mass flux of arsenic and other metals, discharge was determined using the timed volume and float methods^﻿103﻿^. The timed volume method was used for a small stream in the upper part of the basin (no. 15; Fig. 1) where float methods could not be used. In the timed volume method, a graduated container was used along with simultaneous time measurements to determine water volume in the small stream. Measurements were carried out five times and mean discharge was calculated, excluding minimum and maximal values.

The float method was used for streams with higher discharge situated downstream of Złoty Stok in the lower part of the Trująca River basin (nos. 21, 24, 27; Fig. 1). The float method uses water velocity and a cross-section of an area to determine water discharge. Standardised floaters in the regular part of a river channel are used to determine the surface velocity, which is corrected using a roughness coefficient^﻿104﻿,105﻿^. The corrected value is assumed to be the mean water velocity at that point. Although the float method is not widely used in discharge measurements, it is relatively easy to use in small ungauged streams^103^. This method also is particularly useful for measuring streams in the vicinity of Złoty Stok, where water depth (usually less than 0.1 m) makes it difficult to carry out measurements using other methods (e.g. the current meter method).

### Laboratory analyses

#### Water analyses

Measurements of metalloid concentrations in water samples were carried out using graphite furnace atomic absorption spectrometry (GFAAS) for samples with low metal concentrations. Calibration curves were made based on standard solutions (Merck). Measurement errors and RSD% of certified reference material (TMDA-64.3) were 6% and 5%, 5% and 1%, 5% and 3%, and 0.1% and 3% for filterable As, Fe, Al and Zn, respectively.

#### Sediment analyses

Grain-size distribution was determined using a Mastersizer 2000 laser diffractometer^﻿106﻿^. A sediment sample was suspended in a beaker of water with added sodium hexametaphosphate (Calgon). A series of detectors then measured the intensity of light scattered by the particles in the sample for both wavelengths: for larger particles, red (632 nm), and for sub-micron particles, blue light (470 nm). A combination of both wavelength was used to determine sediment fractions ranging from 0.1 to 2000 µm^﻿107﻿^, and sediment fraction classification was based on^﻿108﻿^.

Sediments were dried at 105 ºC for 24 h, then pounded in an agate mortar and sieved to obtain a fraction less than 1 mm. Weighted quantities (~ 1 g) of sediments were digested in a MARS Xpress American microwave (CEM Corporation) in Suprapur 60% HNO_3_. Following dilution in 50 ml of de-ionised water, heavy metals (Zn, Pb, Cu, Cr, Cd, Ni) were determined via GFAAS using an Avanta Sigma GBC atomic absorption spectrometer (Scientific Equipment Ltd.)^﻿109﻿,110﻿^. Standard solutions (Merck) were used for calibration^﻿111﻿^. Blanks were usually below detection limits. The analysis of the metals in the certified reference materials (ISE-934) showed both measurement error and %RSD to be lower than 5%.

The pH(H_2_O) of sediments was measured using distilled water^﻿112﻿,﻿113^. Approximately 10 g of soil was added to a 50-ml beaker and mixed with 25 ml of distilled water. After 24 h, the pH was measured using an Elmetron CX-551 pH-meter.

## Supplementary information

Supplementary Information.

## Data Availability

The datasets generated during and/or analysed during the current study are available from the corresponding author on reasonable request (Lukasz.Stachnik@gmail.com).
