# Supplementary material for: Arsenic pollution in Quaternary sediments and water near a former gold mine
Source: Sci Rep. 2020 Oct 28;10:18458. doi: 10.1038/s41598-020-74403-3 (PMC7595152; doi:10.1038/s41598-020-74403-3)

Supplementary materials to the manuscript: Arsenic pollution in Quaternary sediments and water near a former gold mine (running title: Arsenic in Quaternary sediments and water), Stachnik, Ł.[1]*, Korabiewski B.[1], Raczyk J.[1], Łopuch M.[1], Wieczorek I.[1]
[1] University of Wrocław, Department of Physical Geography, Wrocław, Poland; * - Lukasz.Stachnik@gmail.com

Supplementary Figure S1. Five-year totals of new cancer incidents in gminas of the Ząbkowice Śląskie county for 1986-2015. Data on cancer incidents and inhabitants were obtained from Dolnośląskie Centrum Onkologiczne (Lower Silesia Oncology Center, https://www.dco.com.pl/drn/ accessed on 23 June 2020) and Bank Danych Lokalnych (Local Data Bank, https://bdl.stat.gov.pl/BDL/start, accessed on 23 June 2020), respectively. A) variations between five-years intervals of new cancer incidents B) median of five-years intervals. BAR, CIE, KAZ, STA, ZAS, ZIE, ZST denote Bardo, Ciepłowody, Kamieniec Ząbkowicki, Stoszowice, Ząbkowice Śląskie, Ziębice, and Złoty Stok respectively.

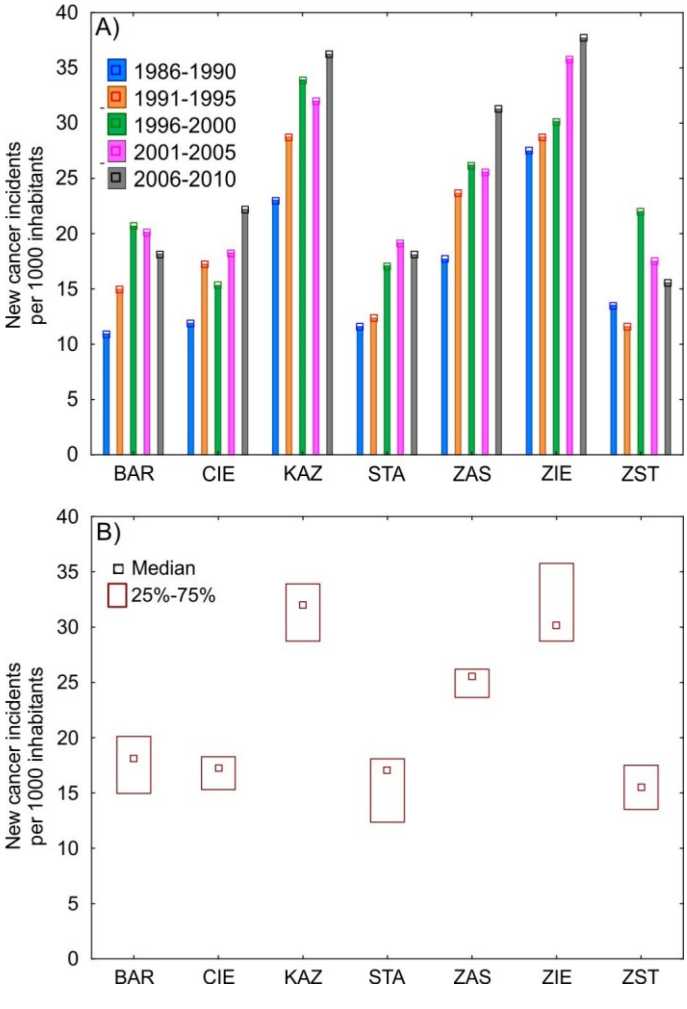

Supplement: Supplementary file 1 — Supplementary Information. [file 41598_2020_74403_MOESM1_ESM.pdf]
